# Supplementary material for: Influence of Intrinsic Physicochemical Properties of Agroforestry Waste on Its Pyrolysis Characteristics and Behavior
Source: Materials (Basel). 2022 Dec 26;16(1):222. doi: 10.3390/ma16010222 (PMC9822187; doi:10.3390/ma16010222)
Supplement: Supplementary file 1 [file materials-16-00222-s001.zip › materials-2033328-supplementary.pdf]

materials

MDPI

Table S1. The minimum, mean and maximum value of *Ea* (kJ·mol⁻¹) obtained by FWO, Friedman, KAS, and Starink methods for RS, ATB, and PS.

Method	RS			ATB			PS		
	Ea[min]	*Ea*[mean]	*Ea*[max]	*Ea*[min]	*Ea*[mean]	*Ea*[max]	*Ea*[min]	*Ea*[mean]	*Ea*[max]
FWO	10.39	167.15	224.07	43.13	195.37	278.62	28.76	200.58	305.15
Friedman	16.07	195.58	250.53	87.06	234.95	309.08	82.51	236.45	320.53
KAS	17.93	177.90	225.96	37.26	207.19	282.83	20.85	191.27	295.24
Starink	11.60	178.60	226.34	37.58	207.57	283.24	22.26	201.53	310.90

Table S2. Variation of Pre-exponential factor (A) from different isoconversional model-free methods (FWO, Friedman, KAS, and Starink) of RS, ATB, and PS

Feedstocks	α	*A* (s^{-1})			
		FWO	Friedman	KAS	Starink
RS	0.10	5.36E-03	2.69E-02	4.42E-02	7.69E-03
	0.15	1.73E+07	3.67E+10	8.55E+06	9.21E+06
	0.20	1.45E+10	1.78E+13	9.74E+09	1.05E+10
	0.25	4.61E+11	9.83E+13	3.56E+11	3.85E+11
	0.30	8.77E+12	1.76E+15	7.64E+12	8.26E+12
	0.35	4.23E+14	1.99E+18	4.39E+14	4.76E+14
	0.40	2.95E+16	1.05E+21	3.74E+16	4.05E+16
	0.45	4.48E+17	5.67E+20	6.40E+17	6.93E+17
	0.50	8.34E+17	5.19E+19	1.21E+18	3.38E+18
	0.55	2.09E+18	2.62E+19	3.12E+18	3.38E+18
	0.60	9.14E+17	3.72E+17	1.28E+18	1.39E+18
	0.65	5.39E+16	6.42E+15	6.29E+16	6.83E+16
	0.70	7.96E+14	2.73E+14	7.25E+14	7.90E+14
	0.75	2.30E+11	4.19E+10	1.25E+11	1.37E+11
	0.80	1.98E+09	7.45E+08	6.98E+08	7.69E+08
ATB	0.10	1.04E+01	1.12E+05	2.86E+00	3.07E+00
	0.15	1.95E+04	4.15E+08	7.54E+03	8.10E+03
	0.20	1.27E+07	3.75E+11	6.58E+06	7.07E+06
	0.25	5.80E+08	5.58E+11	3.53E+08	3.80E+08
	0.30	1.51E+10	6.20E+12	1.05E+10	1.14E+10
	0.35	6.49E+11	8.43E+14	5.33E+11	5.75E+11
	0.40	7.70E+13	5.32E+17	7.86E+13	8.49E+13
	0.45	2.99E+15	2.08E+18	3.58E+15	3.87E+15
	0.50	4.25E+17	2.70E+21	6.40E+17	6.92E+17
	0.55	1.59E+20	6.66E+24	3.16E+20	3.43E+20
	0.60	4.37E+21	2.76E+24	1.01E+22	1.09E+22

	0.65	6.48E+21	3.06E+22	1.50E+22	1.62E+22
	0.70	4.15E+21	4.06E+21	9.18E+21	9.96E+21
	0.75	3.57E+21	6.04E+21	7.65E+21	8.31E+21
	0.80	1.48E+21	9.07E+19	2.92E+21	3.18E+21
PS	0.10	3.45E-01	2.75E+04	5.55E-02	7.75E-02
	0.15	7.11E+01	7.01E+06	1.21E+01	2.13E+01
	0.20	1.24E+05	3.52E+10	2.12E+04	5.34E+04
	0.25	1.69E+08	1.73E+13	2.88E+07	1.03E+08
	0.30	8.24E+10	3.51E+15	1.38E+10	6.71E+10
	0.35	1.08E+13	6.60E+15	1.78E+12	1.11E+13
	0.40	1.82E+14	1.89E+16	2.94E+13	2.11E+14
	0.45	3.72E+15	1.30E+18	5.91E+14	4.92E+15
	0.50	1.90E+17	4.41E+20	2.97E+16	3.01E+17
	0.55	9.70E+18	6.56E+21	1.49E+18	1.84E+19
	0.60	1.04E+21	2.47E+24	1.58E+20	2.47E+21
	0.65	2.24E+22	5.12E+24	3.34E+21	6.12E+22
	0.70	2.61E+23	4.00E+25	3.83E+22	7.94E+23
	0.75	2.09E+23	3.28E+23	3.02E+22	6.19E+23
	0.80	7.21E+22	2.69E+22	1.02E+22	1.99E+23

Note: Results calculated at the heating rate 10 °C·min^{-1}
